# Phosphatidylcholine PC ae C44:6 in cerebrospinal fluid is a sensitive biomarker for bacterial meningitis

**DOI:** 10.1186/s12967-019-02179-w

**Published:** 2020-01-07

**Authors:** Leonardo Silva de Araujo, Kevin Pessler, Kurt-Wolfram Sühs, Natalia Novoselova, Frank Klawonn, Maike Kuhn, Volkhard Kaever, Kirsten Müller-Vahl, Corinna Trebst, Thomas Skripuletz, Martin Stangel, Frank Pessler

**Affiliations:** 1 Research Group “Biomarkers for Infectious Diseases”, TWINCORE Centre for Experimental and Clinical Infection Research, Feodor-Lynen-Str. 7, 30625 Hannover, Germany; 2 Helmholtz Centre for Infection Research, Brunswick, Germany; 3 Division of Bioinformatics, United Institute of Informatics Problems, Minsk, Belarus; 4 Research Core Unit Metabolomics, Hannover Medical School, Hannover, Germany; 5 Department of Psychiatry, Social Psychiatry and Psychotherapy, Hannover Medical School, Hannover, Germany; 6 Clinical Neuroimmunology and Neurochemistry, Dept. of Neurology, Hannover Medical School, Hannover, Germany; 7 Center for Systems Neuroscience, Hannover, Germany; 8 Cluster_of_Excellence RESIST (EXC 2155), Hannover Medical School, Hannover, Germany; 9 Centre for Individualised Infection Medicine, Hannover, Germany; 10 Present Address: Molecular and Experimental Mycobacteriology, Research Center Borstel-Leibniz Lung Center, Sülfeld, Germany

**Keywords:** Biomarker, Brain, Diagnosis, Encephalitis, Infection, Lecithin, Lipids, Lipidomics, Meningitis, Metabolomics

## Abstract

**Background:**

The timely diagnosis of bacterial meningitis is of utmost importance due to the need to institute antibiotic treatment as early as possible. Moreover, the differentiation from other causes of meningitis/encephalitis is critical because of differences in management such as the need for antiviral or immunosuppressive treatments. Considering our previously reported association between free membrane phospholipids in cerebrospinal fluid (CSF) and CNS involvement in neuroinfections we evaluated phosphatidylcholine PC ae C44:6, an integral constituent of cell membranes, as diagnostic biomarker for bacterial meningitis.

**Methods:**

We used tandem mass spectrometry to measure concentrations of PC ae C44:6 in cell-free CSF samples (n = 221) from patients with acute bacterial meningitis, neuroborreliosis, viral meningitis/encephalitis (herpes simplex virus, varicella zoster virus, enteroviruses), autoimmune neuroinflammation (anti-NMDA-receptor autoimmune encephalitis, multiple sclerosis), facial nerve and segmental herpes zoster (shingles), and noninflammatory CNS disorders (Bell’s palsy, Tourette syndrome, normal pressure hydrocephalus).

**Results:**

PC ae C44:6 concentrations were significantly higher in bacterial meningitis than in all other diagnostic groups, and were higher in patients with a classic bacterial meningitis pathogen (e.g. *Streptococcus pneumoniae, Neisseria meningitidis, Staphylococcus aureus*) than in those with less virulent or opportunistic pathogens as causative agents (P = 0.026). PC ae C44:6 concentrations were only moderately associated with CSF cell count (Spearman’s ρ = 0.45; P = 0.009), indicating that they do not merely reflect neuroinflammation. In receiver operating characteristic curve analysis, PC ae C44:6 equaled CSF cell count in the ability to distinguish bacterial meningitis from viral meningitis/encephalitis and autoimmune CNS disorders (AUC 0.93 both), but had higher sensitivity (91% vs. 41%) and negative predictive value (98% vs. 89%). A diagnostic algorithm comprising cell count, lactate and PC ae C44:6 had a sensitivity of 97% (specificity 87%) and negative predictive value of 99% (positive predictive value 61%) and correctly diagnosed three of four bacterial meningitis samples that were misclassified by cell count and lactate due to low values not suggestive of bacterial meningitis.

**Conclusions:**

Increased CSF PC ae C44:6 concentrations in bacterial meningitis likely reflect ongoing CNS cell membrane stress or damage and have potential as additional, sensitive biomarker to diagnose bacterial meningitis in patients with less pronounced neuroinflammation.

## Introduction

Initiating antibiotic treatment as early as possible is important to optimize clinical outcome of bacterial meningitis [1, 2]. However, the diagnosis and treatment of bacterial meningitis continues to present challenges, as causative pathogens may not be detected in all patients in a timely manner and results of routine cerebrospinal fluid (CSF) parameters such as leukocyte count are often insufficiently sensitive and specific, for instance in patients with comorbidities or atypical pathogens as causative agents [3]. In potentially organ- or life-threatening diseases such as bacterial meningitis it is important to avoid undertreatment; a clinically robust diagnostic should therefore combine high sensitivity (i.e. a high percentage of cases has a positive test result) and negative predictive value (i.e. a negative test can reliably rule out the presence of disease; NPV).

Emerging evidence suggests that measuring concentrations of small molecules in CSF can help to identify CSF biomarkers for various aspects of central nervous system (CNS) infections such as differentiating between infectious and autoimmune etiologies [4], assessing CNS complications of chronic infections [5, 6], or detecting CNS extension of infections with a presumed primary site outside of the CNS [7]. We have recently shown that major changes in CSF metabolite populations occur in viral CNS infections [4, 7, 8] and that certain membrane phospholipids, when measured in cell-free CSF, constitute highly accurate CSF biomarkers for meningoencephalitis during varicella zoster virus (VZV) reactivation [7] and for a diagnosis of enterovirus meningitis even in patients with normal CSF cell counts [8]. However, these analyses also showed that in virally infected, autoimmune, or non-inflamed samples many metabolites including phospholipids were present in only low concentrations, raising the hypothesis that some of them may be selectively more abundant in CSF from bacterial meningitis and may therefore constitute biomarkers for this challenging/life-threatening infectious disease.

Phosphatidylcholines (PC) are a class of phospholipids that possess a choline head group and two fatty acids (or one fatty acid and one fatty alcohol) linked to a glycerol phosphoric acid backbone [9]. Due to their bipolar nature they are found as ubiquitous structural constituents of the lipid bilayer of eukaryotic cell membranes, but they also fulfill a variety of regulatory functions in that the action of enzymes such as phospholipases can lead to the release of products that mediate intracellular signals [9]. Functionally, PC and phospholipase activity have been linked to many processes that underlie inflammation and cell stress or damage, all of which also become potentially active during CNS infections and may thus reflect interactions between pathogens and brain parenchyma and/or immune cells. Of note, PC are considered critical players in the balance between cell survival and death [10], which has clear implications for pathogenesis and outcome of bacterial CNS infections, as they may feature a high degree of damage to brain parenchyma, leading to long-term clinical sequelae. We have, therefore, analyzed data from a targeted metabolomic screen of 221 CSF samples to identify membrane phospholipid biomarkers that are preferentially more abundant in CSF from patients with bacterial meningitis.

## Participants, materials, and methods

### Study population and biosamples

The study was conducted according to the Declaration of Helsinki and was approved by the Ethics Committee of Hannover Medical School (file no. 2413-2014). Recruitment of patients, processing of CSF and sociodemographic and standard laboratory diagnostic data of the diagnostic groups are also described in [4, 7, 8]. Briefly, CSF was obtained during clinically indicated lumbar puncture and processed within 2 h. The following standard diagnostic CSF parameters were analyzed directly after lumbar puncture: leukocyte count (counted manually with a Fuchs-Rosenthal counting chamber), protein concentration (Bradford dye-binding assay), lactate concentration (photometric assay), Q-albumin ratio (albumin concentration in CSF/albumin concentration in serum), IgG-index (IgG concentration in CSF/IgG concentration in serum divided by Q-albumin ratio; age-adjusted reference limit = 4 + (age/15). IgG and albumin were measured in CSF and serum in the same latex-enhanced assay by kinetic nephelometry (Beckman Coulter IMMAGE). Blood C-reactive protein (CRP) levels and complete blood counts with differential were determined in the clinical diagnostic lab of Hannover Medical School. Aliquots of cell-free CSF were obtained by centrifugation and kept frozen at − 80 °C until the metabolomic analyses. The samples for the present study (N = 221) were retrospectively selected and comprised the following diagnoses: bacterial meningitis (BacM, n = 32), *Borrelia burgdorferi* neuroborreliosis (Borrelia, n = 34), herpes simplex encephalitis (HSE, n = 9), VZV meningoencephalitis (VZV ME, n = 15), enterovirus meningitis (EntM, n = 10), facial zoster (VZV fac, n = 16), segmental zoster (VZV seg, n = 14; also known as shingles), anti-NMDA-receptor autoimmune encephalitis (NMDA, n = 8), multiple sclerosis (MS, n = 17), Bell’s palsy (Bell, n = 11), Gilles de la Tourette syndrome (GTS, n = 20), and normal pressure hydrocephalus (NPH, n = 35). The standard laboratory parameters of the enterovirus meningitis specimens are derived from a larger cohort study on this entity [11]. Case definitions (diagnostic criteria) are summarized in Additional file 1: Table S1, sociodemographic and standard clinical laboratory parameters in Additional file 2: Table S2, and the most likely causative pathogens isolated from the bacterial meningitis patients in Table 1.

**Table 1:** Causative pathogens in 32 patients with bacterial meningitis

Pathogen	N (%)
Streptococci	
*S. pneumoniae*	12 (36)
*S. pyogenes*	1 (3.0)
*S. salivarius*^a^	1 (3.0)
*S. anginosus*^a^	1 (3.0)
Staphylococci	
*S. aureus*	3 (9.1)
*S. epidermidis*^a^	2 (6.1)
*S. hominis*^a^	1 (3.0)
*S. warneri*^a^	1 (3.0)
Others	
*N. meningitides*	3 (9.1)
*Listeria monocytogenes*	3 (9.1)
*H. influenzae*	1 (3.0)
*E. coli*	1 (3.0)
*Micrococcus luteus*^a^	1 (3.0)
*Peptostreptococcus* sp.^a^	1 (3.0)
*Bacillus* sp.^a^	1 (3.0)

N= 33 isolates due to coinfection. Additional coinfections (second pathogens not included in the analysis): *S. pneumoniae*/HSV-2, *L. monocytogenes*/*Borrelia burgdorferi*

^a^Pathogen commonly considered opportunistic and treated as such in Fig. 2

### Measurement of PC ae C44:6 concentrations in CSF by mass spectrometry

The concentration values for PC ae C44:6 were taken from a larger data set of metabolomic CSF analysis, obtained by high-performance liquid chromatography tandem mass spectrometry (HPLC MS/MS) and direct flow injection MS/MS with the AbsoluteIDQ™ p180 kits (Biocrates Life Sciences, Innsbruck, Austria). This kit allows the quantification of 188 analytes, comprising 42 amino acids and amino acid metabolites, 91 glycerophospholipids, 15 sphingolipids, 40 acylcarnitines, and the sum of hexoses. Details of the measurement procedure are described in [7]. Other aspects of the resulting data set, which do not include PC ae C44:6, have been published separately [4, 7, 8]. References [7, 8] feature comprehensive analyses of the data set, but PC ae C44:6 was excluded from those analyses due to the high frequency of concentrations below limit of detection (LOD) in the samples other than bacterial meningitis. The LOD of PC ae C44:6 was determined to be 9 nM, and all values < LOD were replaced with the pseudo-value of LOD/2 ≈ 5 nM.

### Statistical analyses

PC ae C44:6 concentrations in CSF were non-normally distributed across the 221 samples. Spearman’s rank correlation coefficient (ρ) was therefore used for correlation analyses and the Mann–Whitney U test for significance (P < 0.05) of between-group differences in medians. Chi squared (*Χ*^2^) test and Fisher’s exact test were used to assess significance of differences in categorical variables. Receiver operating characteristic (ROC) curve analysis was used to quantify the discriminatory accuracy of biomarkers. A perfect biomarker has an area under the curve (AUC) of 1.0, and the robustness of the curve is further supported by asymptotic P values of < 0.05 and lower bound 95% confidence intervals not crossing the chance-line of 0.5. Analyses were carried out using GraphPad PRISM v.8 (GraphPad Software, Inc.) and the open source software MetaboAnalyst (http://www.metaboanalyst.ca).

## Results

### Elevated PC ae C44:6 concentrations in CSF from patients with bacterial meningitis

**Fig. 1:**
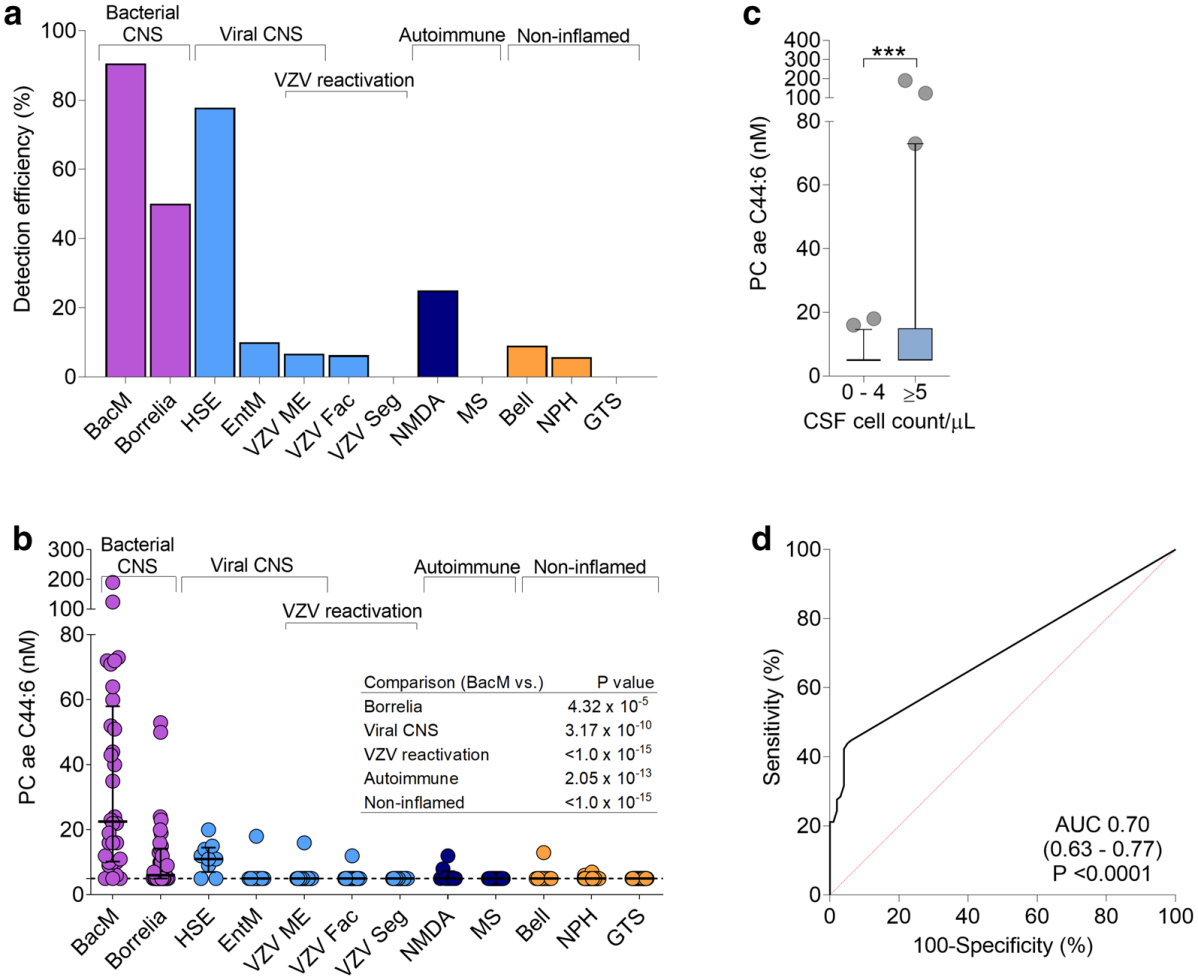
PC ae C44:6 concentrations in CSF are highly elevated in bacterial meningitis. Concentrations were measured by mass spectrometry using cell-free CSF in samples from patients with bacterial meningitis (BacM, n = 32), *Borrelia burgdorferi* neuroborreliosis (Borrelia, n = 34), HSV encephalitis (HSE, n = 9), varicella zoster virus meningoencephalitis (VZV ME, n = 15), enterovirus meningitis (EntM, n = 10), facial zoster (VZV fac, n = 16), segmental zoster (VZV seg, n = 14), anti-NMDA-receptor autoimmune encephalitis (NMDA, n = 8), multiple sclerosis (MS, n = 17), Tourette syndrome (GTS, n = 20), Bell’s palsy (Bell, n = 11), and normal pressure hydrocephalus (NPH, n = 35). **a** Detection efficiency (% of samples with concentrations > LOD) of PC ae C44:6 in the 12 diagnoses. Detection rate was by far the highest in bacterial meningitis. **b** PC ae C44:6 concentrations across the 12 diagnoses. Median concentrations were highest in bacterial meningitis, but a considerable spread of values is evident within this group. **c** Higher median PC ae C44:6 concentrations in samples with CSF cell ≥ 5/μL. The boxes span the interquartile range (25–75th percentile), the circles define outlying values > 97.5th percentile. ***P < 0.001. **d** ROC analysis comparing PC ae C44:6 concentrations in samples with CSF cell count of 0–4 and ≥ 5/μL, demonstrating only a moderate association with neuroinflammation

**Fig. 2:**
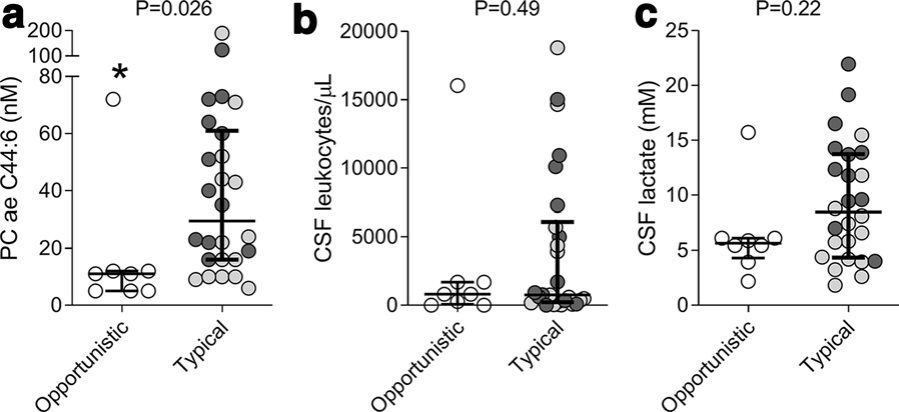
Higher PC ae C44:6 CSF concentrations in meningitis caused by bacteria typically associated with bacterial meningitis. The pathogens were divided into two groups according to expected virulence, pathogens typically associated with meningitis (“Typical”) and “Opportunistic” pathogens as indicated in Table 1. P values for between-group differences in median values were determined with the Mann–Whitney U test (two-tailed) and are shown in the figure panels. **a** PC ae C44:6 concentration. **b** CSF cell count. **c** CSF lactate concentration. Open circles: opportunistic pathogens; grey and black circles: typical pathogens, of which the black circles refer to *S. pneumoniae* only. In case of the two coinfections, both pathogens are indicated with separate symbols

Within the entire data set comprising 188 analytes, we searched for analytes whose measured concentrations were > LOD preferentially in bacterial meningitis compared to the noninfected/noninflamed samples. This analysis revealed two analytes, PC ae C44:6 and kynurenine (P < 0.005, Fisher’s exact test). Subsequent detailed analysis of kynurenine identified it as a biomarker for both bacterial and viral CNS infections [4]. As shown in Fig. 1a, PC ae C44:6 was detected > LOD in nearly all bacterial meningitis specimens and half of the neuroborreliosis samples. With the notable exception of HSE, detection efficiency was very low in all of the other samples. Measured absolute PC ae C44:6 concentrations followed a similar trend and were significantly higher in bacterial meningitis than in the other 11 diagnoses (Fig. 1b), with fold differences varying from 3.3 (compared with neuroborreliosis) to 7.7 (vs. multiple sclerosis and Tourette syndrome). Even though median PC ae C44:6 concentrations were significantly higher in inflamed (leukocyte count ≥ 5 cells/μL) than in non-inflamed (0–4 cells/μL) samples (Fig. 1**c**), ROC analysis revealed only a moderate association between increased PC ae C44:6 concentrations and neuroinflammation (Fig. 1d), and there were six samples with normal cell counts but elevated PC ae C44:6 concentrations. Lastly, we tested whether PC ae C44:6 concentrations differed according to the causative bacterial pathogen. The three highest PC ae C44:6 concentrations were measured in patients with *S. aureus* and *S. pneumoniae* (n = 2) infection, whereas the three lowest occurred in infections with pathogens not typically associated with CNS infections (*Bacillus* sp., *Staphylococcus warneri, Micrococcus luteus*). A more quantitative comparison to determine differences in PC ae C44:6 concentrations by pathogen was clearly limited by the small group sizes. We therefore divided the pathogens into two groups according to overall pathogenicity, i.e. virulent bacteria commonly associated with meningitis (“Typical”) and less virulent bacteria requiring an immune compromise or exogenous factors such as indwelling hardware to cause invasive infection (“Opportunistic”). PC ae C44:6 concentrations were significantly higher in the “Typical” group, whereas values of the classic CSF markers of neuroinflammation cell count and lactate concentration did not differ between the two groups (Fig. 2).

### Discrepancies between PC ae C44:6 concentrations and standard blood and CSF parameters of inflammation

**Fig. 3:**
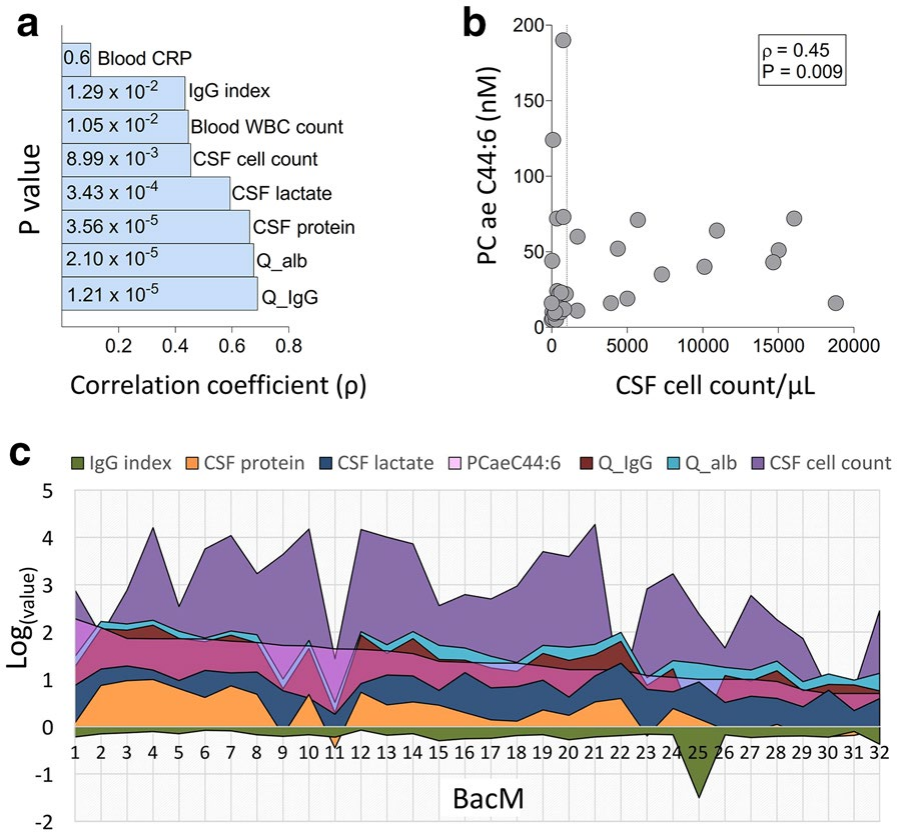
Correlations and discrepancies between PC ae C44:6 concentrations and standard blood and CSF parameters of inflammation. **a** Correlations between PC ae C44:6 concentration and the standard blood and CSF parameters in bacterial meningitis. X-axis values correspond to Spearman’s ρ, the values inside the bars to P values. **b** Scatter plot of PC ae C44:6 concentrations vs. CSF cell count in bacterial meningitis, revealing several samples with high PC ae C44:6 concentrations but low cell counts and vice versa. **c** Comparisons of PC ae C44:6 concentration and the six standard CSF parameters (plotted on the y-axis, log (10) transformed) across the 32 bacterial meningitis samples. The samples are arranged along the x-axis in descending order of PC ae C44:6 concentration

A correlation analysis with the eight standard diagnostic blood and CSF parameters showed that, in bacterial meningitis, PC ae C44:6 concentrations correlated most strongly with Q-IgG, Q-albumin and CSF protein concentration, i.e. parameters associated with dysfunction of the blood-CSF-barrier (BCB) and, in the case of protein concentration, potentially also tissue damage (Fig. 3a). Consistent with the only modest correlation between PC ae C44:6 concentrations and the standard parameters including CSF cell count, a scatter plot revealed several bacterial meningitis samples with high PC ae C44:6 concentrations but relatively low cell counts and vice versa (Fig. 3b). As shown in Fig. 3c, there were discrepancies between PC ae C44:6 concentrations and each of the standard parameters (i.e. high PC ae C44:6 value but low standard parameter value, or vice versa) in several patients. Taken together, these results suggest that in selected patients PC ae C44:6 concentrations measure features of disease that are not reflected by the standard parameters in a pattern that various from patient to patient.

### PC ae C44:6 is an accurate CSF biomarker for bacterial meningitis

We then used ROC curve analysis to assess the diagnostic accuracy of PC ae C44:6. Since neuroborreliosis is not in the first line differential diagnosis of acute bacterial meningitis, we focused on the clinically more relevant comparison bacterial meningitis vs. viral CNS infections and autoimmune neuroinflammation. PC ae C44:6 accurately differentiated bacterial meningitis from the non-bacterial diagnoses with the same AUC of 0.93 as CSF cell count, but differed from cell count by an overall higher accuracy at the trade-off value (i.e. the point on the ROC curve where the sum of sensitivity + specificity is maximal) and a markedly higher sensitivity and NPV, but lower specificity and positive predictive value (PPV) (Table 2).

**Table 2:** Comparison of diagnostic accuracy of PC ae C44:6 and CSF cell count to distinguish between acute bacterial meningitis (n = 32) and nonbacterial infectious, autoimmune, and non-inflammatory CNS disorders (n = 189)

Parameter	AUC (95% CI)	Cut-off	Accuracy^a^	Sensitivity	Specificity	PPV	NPV	Fold difference^b^
CSF cell count	0.93^c^ (0.89–0.97)	> 1000 cells/μL	70.0	40.6	99.4	92.9	89	64.7
PC ae C44:6	0.93^c^ (0.87–0.99)	> 5 nM	90.5	90.6	90.3	65.9	97.9	6.8

Values were obtained by ROC analysis. Sensitivity, specificity, positive and negative predictive value (PPV and NPV) were determined at the trade-off value (Youden index), corresponding to the maximal value of (sensitivity + specificity)/2 in the case of PCaeC44:6 and the clinically recommended cut-off for cell count. Diagnostic groups included in the analysis: bacterial meningitis (n = 32), HSV encephalitis (n = 9), varicella zoster virus meningoencephalitis (n = 15), enterovirus meningitis (n = 10), facial zoster (n = 16), segmental zoster (n = 14), anti-NMDA-receptor autoimmune encephalitis (n = 8), multiple sclerosis (n = 17), Tourette syndrome (n = 20), Bell’s palsy (n = 11), and normal pressure hydrocephalus (n = 35)

*AUC* area under the curve, *CI* confidence interval, *ROC* receiver operating characteristic

^a^Defined as (sensitivity + specificity)/2 at the cut-off value, ^b^ratio of mean values, bacterial meningitis/all others, ^c^asymptotic significance, P < 0.001

### PC ae C44:6 improves sensitivity and NPV to diagnose bacterial meningitis in patients with a low degree of neuroinflammation

**Fig. 4:**
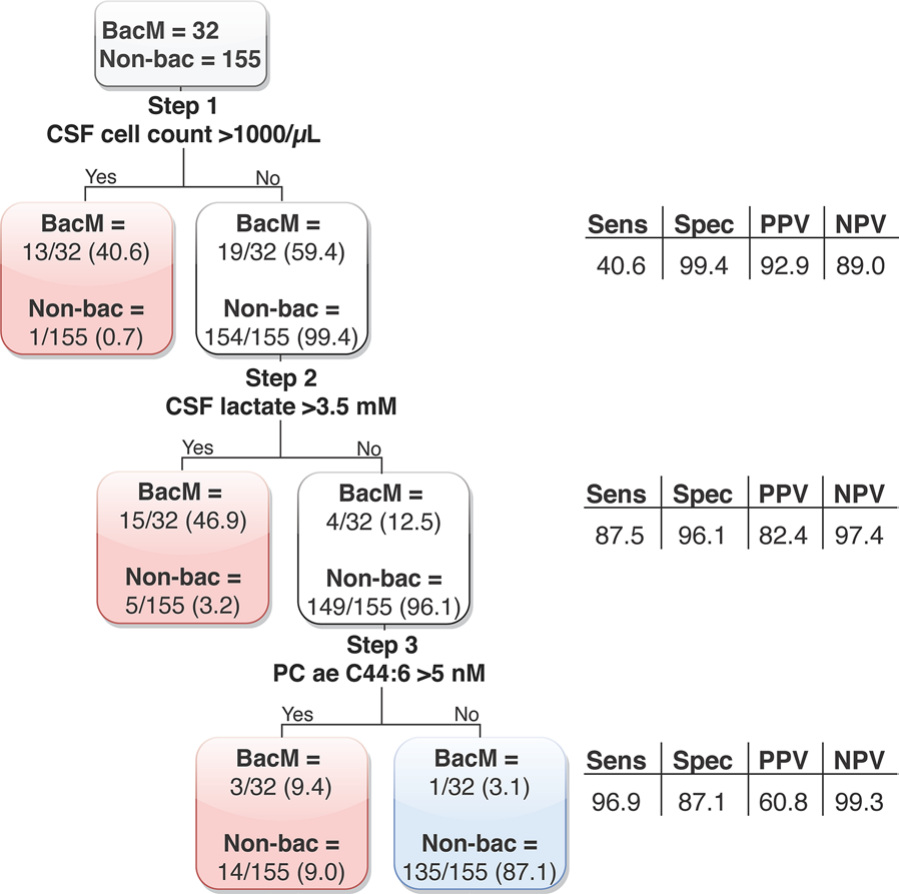
Diagnostic algorithm revealing improved diagnostic sensitivity by including PC ae C44:6. 187 CSF samples (bacterial meningitis, n = 32; HSV encephalitis, n = 9; varicella zoster virus meningoencephalitis, n = 15; enterovirus meningitis, n = 10; facial zoster, n = 16; segmental zoster, n = 14; anti-NMDA-receptor autoimmune encephalitis, n = 8; multiple sclerosis, n = 17; Tourette syndrome, n = 20; Bell’s palsy, n = 11; and normal pressure hydrocephalus, n = 35) were subjected to a progressive classification scheme based on CSF cell count and lactate levels, using the commonly used cut-off values for bacterial meningitis [12] indicated in the figure. Adding PC ae C44:6 led to the correct identification of 3 of 4 samples which had been misclassified due to atypically low neuroinflammation

To test whether PC ae C44:6 concentrations could improve a diagnostic algorithm, we performed a classification-tree analysis based on cut-off values of CSF leukocyte count (1000 cells/μL) and lactate (3.5 mM) for the diagnosis of bacterial meningitis, as recommended by the German Society for Neurology [12] (Fig. 4). As expected, these two well-validated parameters correctly classified the great majority (28/32 = 88%) of bacterial meningitis cases. However, a PC ae C44:6 concentration of > 5 nM led to an additional correct classification of three of the four previously misclassified bacterial meningitis cases, all of which had been misclassified because of cell counts and lactate levels below the cut-off values. However, this also led to an additional 14 false positives that were misclassified as bacterial meningitis, demonstrating that the gain in sensitivity (from 88 to 97%) and NPV (97 to 99%) came at the cost of a loss in specificity (from 96 to 87%) and PPV (from 82 to 61%). It should be noted that the one bacterial meningitis patient who was not diagnosed correctly by any of these three parameters harbored infection with a pathogen of low virulence (*Staphylococcus anginosus*).

## Discussion

Following our previous results of increased CSF concentrations of free phospholipids in viral CNS infections compared to disease controls without CNS infection [7, 8], we tested the hypothesis that selected CSF phospholipid species are preferentially abundant also in bacterial meningitis. For this purpose, we analyzed a targeted metabolomic data set comprising 221 CSF samples spanning bacterial, viral, and non-infectious inflammatory and noninflammatory diagnoses and identified elevated PC ae C44:6 concentration as an accurate biomarker to differentiate bacterial meningitis from viral CNS infections and autoimmune neuroinflammation. A finding of particular clinical relevance was the high sensitivity of PC ae C44:6 to detect bacterial meningitis patients with unusually mild neuroinflammation.

### Usefulness of PC ae C44:6 in identifying bacterial meningitis patients with low CSF leukocyte counts and lactate levels

The diagnostic algorithm (Fig. 4) underscored the clinical value of the standard CSF parameters cell count and lactate, but it also suggested that the added value of PC ae C44:6 may lie in identifying cases of bacterial meningitis whose standard CSF parameters do not strongly support this diagnosis due to a low degree of neuroinflammation. A clinical scenario in which this is particularly important is the evaluation of the immunocompromised patient, as clinical presentations may be atypical and may be particularly difficult to differentiate from non-infectious CNS syndromes, and diminished numbers or function of inflammatory cells may skew results of standard CSF diagnostic parameters [3]. The human cost of undertreating acute bacterial meningitis (i.e. a poor clinical outcome) is high. The gain in sensitivity and NPV which resulted from integrating PC ae C44:6 into the diagnostic algorithm is therefore of high clinical relevance. We have recently described that a combined classifier consisting of PC ae C36:3, PC ae C36:5 and PC ae C38:5 distinguished perfectly between patients with enterovirus meningitis and normal CSF cell counts and non-inflamed control samples [8]. It remains to be demonstrated in additional cohorts whether it is a general property of PCs to identify infected samples with low or normal cell counts, but it is tempting to speculate that they reflect cell-damaging interactions between pathogens and host cells and that the involved PCs may at least partially differ according to the pathogen or pathogen class and thus facilitate the clinically highly important distinction between bacterial and viral etiologies.

### Potential causes of elevated PC ae C44:6 concentrations

Since we analyzed cell-free CSF, it is difficult to ascertain the source and mechanisms of the elevated PC ae C44:6 concentrations. One possible mechanism would be increased entry into the CNS due to dysfunction of the BCB, which would agree with the observed positive correlation between Q-albumin and PC ae C44:6 levels. However, PC concentrations in peripheral blood usually decrease during acute inflammation, possibly due to formation of complexes with CRP and subsequent removal in the reticuloendothelial system [13] or increased catabolism by phospholipases [14]. We therefore do not think that increased import through the BCB alone would account for the observed increased concentrations in CSF. Another possibility would be increased synthesis in the cytidine diphosphate-choline cycle, which is active across mammalian organs including brain and in neuronal cell lines [15]. This would then be followed by release from viable cells, for instance as part of microvesicles and exosomes. Alternatively, a block to catabolism to lysoPC by phospholipase A2 (which also occurs as a secretory form in CSF [16]) would be conceivable, but we consider this highly unlikely, as we have actually detected elevated levels of lysoPC in CSF from patients with VZV meningoencephalitis [7], and there is no pathogenetically plausible reason why this should be different in bacterial meningitis. The most plausible explanation is that the PC is released from damaged and/or dying cells, as substantial damage to brain parenchyma is a common feature of bacterial meningitis. This would also agree well with the observation that among the three viral CNS infections studied herein, the highest PC ae C44:6 concentrations were measured in the form with the greatest risk of CNS tissue damage, i.e. HSE. The presence of several patients with strong discrepancies between PC levels and cell counts and the overall only moderate correlation between PC and cell count also clearly suggest that the elevated PC levels are not just a manifestation of neuroinflammation, particularly not of mere accumulation of inflammatory cells in the CNS. Very little is known about the relative distribution of the many types of PC across different cell types in the CNS; it is therefore currently not possible to discern whether the PC ae C44:6 originates from specific cell types. Thus, it would now be of great interest to test the hypothesis that the pattern of released membrane phospholipids might reflect the identity of the damaged cell and the severity of parenchymal damage in bacterial meningitis, and that it could thus be used to improve early prognosis of clinical outcome.

### Biochemical identity of PC ae C44:6

It is important to note that, in the mass-spectrometry assay used here, the mass reported as PC ae C44:6 can correspond to either one of the two isobars PC O-44:6 and PC 43:6 (of which only the former contains a fatty alcohol derivative), each of which can comprise several isomers. Thus, it is premature to assign more specific mechanistic implications of increased PC ae C44:6 concentrations. It is noteworthy, though, that at least one of the described isomers, 1-alkyl-2-arachidonyl-*sn*-glycerol-3-phosphate, contains arachidonic acid (C20:4) [17]. This could be released by the action of phospholipase A2, which could modulate inflammation by further catabolism to prostaglandins and other eicosanoids. The net effect would depend on the balance between pro- and anti-inflammatory eicosanoids.

## Limitations and strengths of this study

This study is limited by the small sample sizes in some of the groups, in particular it would have strengthened the study if more HSE samples had been available, as this group clearly had higher PC ae C44:6 concentrations than the other two viral CNS infections, probably due to the clinically well documented higher degree of parenchymal damage. In addition, the inability to tease out the isomers making up PC ae C44:6 limits the biological interpretation of our results. On the other hand, clear strengths of the study are the availability of detailed clinical data, case definitions according to standard criteria, processing of CSF samples by the treating clinicians within a narrow time window and according to unified protocols [4], and the use of a broadly validated robust mass-spectrometric measuring system [18].

## Conclusions

In summary, this comprehensive analysis in over 200 CSF samples demonstrates a close association of high concentrations of PC ae C44:6 with bacterial meningitis and reveals its value as an additional diagnostic biomarker, mostly due to its ability to correctly identify patients with an unusually low degree of neuroinflammation.

## Supplementary information

**Additional file 1: Table S1.** Diagnostic criteria and clinical information.

**Additional file 2: Table S2.** Demographic and clinical laboratory characteristics.

## Data Availability

The data used and/or analyzed are available from the corresponding author on a reasonable request.
