# Supplementary material for: Phosphatidylcholine PC ae C44:6 in cerebrospinal fluid is a sensitive biomarker for bacterial meningitis
Source: J Transl Med. 2020 Jan 7;18:9. doi: 10.1186/s12967-019-02179-w (PMC6945415; doi:10.1186/s12967-019-02179-w)
Supplement: Supplementary file 1 — Additional file 1: Table S1. Diagnostic criteria and clinical information. [file 12967_2019_2179_MOESM1_ESM.docx]

| **Table S1.** Diagnostic criteria and clinical information* | | | |
| --- | --- | --- | --- |
| **Diagnosis** | **Criteria** | **Disease grade/activity** | **Systemic treatment at time of lumbar puncture ^a^** |
| Bacterial meningitis (n=32) | Clinical meningitis, pleocytosis (≥5 cells/μL), detection of bacterial pathogen in CSF or blood ^b^ | Detected pathogens:  *See Table 1* | Antibiotics (n=4)  - ceftriaxone, ampicillin  - ciprofloxacin, metronidazole  - ceftriaxone, cephazolin  - gentamycin, ampicillin, ceftazidime |
| Neuroborreliosis (n=34) | Neurological deficit and inflammatory CSF syndrome; intrathecal synthesis of *Borrelia* IgG or elevated *Borrelia* specific antibody index ^c^ | Second-stage neuroborreliosis (100%), duration of symptoms ≤6 months | Antibiotics (n=6)  ceftriaxone  - in combination with  acyclovir (n=2)  corticosteroids (n=1)  acyclovir and corticosteroids (n=1) |
| HSV encephalitis (n=9) | Mental status changes and positive HSV PCR or elevated (>1.5) ASI |  | Acyclovir (n=4)  - with ampicillin (n=1) |
| VZV meningitis/encephalitis (n=15) | Detection of VZV in CSF by PCR and/or intrathecal synthesis of VZV IgG,  clinical meningitis/encephalitis with or without typical zoster rash | Meningitis, 73%  Encephalitis, 27% | Immunosuppression (n=1)  -rituximab, bendamustin (3weeks prior to lumbar puncture due to mantle cell lymphoma) |
| Enterovirus meningitis (n=10) | Clinical meningitis and detection of enterovirus in CSF by PCR | Acute onset (100%)  (symptoms ≤3 months) | - |
| Facial nerve zoster (n=16) | Facial palsy with or without typical zoster rash, detection of VZV DNA in CSF by PCR and/or intrathecal synthesis of VZV IgG |  | - |
| Segmental zoster (n=14) | Typical segmental zoster skin rash, and/or detection of VZV DNA in CSF by PCR and/or intrathecal synthesis of VZV IgG |  | - |
| Anti-NMDA-R encephalitis (n=8) | Clinical encephalitis and detection of IgG anti-NMDA-R antibodies in CSF | Acute onset (100%)  (symptoms ≤3 months) | - |
| Multiple sclerosis (n=17)  -Relapsing remitting (n=15)  -Secondary progressive (n=2) | McDonald 2017 criteria ^d^ | Oligoclonal bands CSF (100%)  Acute flare/relapse (82%)  MRI gadolinium enhancing lesions (70%)  Stable (12%) | Corticosteroids (n=2) |
| Tourette syndrome  (n=20) | Criteria according to DSM-5 ^e^ | Classified by YGTSS-TTS  mild (10%)  moderate (70%)  severe (20%) | Symptomatic treatment (n=3)  -Abilify (n=1)  -Dronabinol (n=1)  -Sativex (n=1) |
| Bell’s palsy  (n=11) | Facial nerve palsy without evidence of infectious etiology or pleocytosis |  | - |
| Normal pressure hydrocephalus  (n=35) | Normal CSF pressure, typical findings on CT or MRI, at least one symptom of Hakim triad ^f^ |  | - |
| *Adapted from ref. 4 (Sühs et al. J Infect Dis 2019; 220:127-138) with kind permission by the publisher.  ^a^ Excluding antipyretics, analgesics and medications for unrelated conditions  ^b^ Brouwer, M.C, Thwaites, G.E., Tunkel A.R, van de Beek, D. Lancet. 380 (2012) 1684-1692.  ^c^ Koedel, U., Fingerle, V. & Pfister, H.W. Nat. Rev. Neurol 2015; 11, 446-456  ^d^ Thompson, A et al., Diagnosis of multiple sclerosis: 2017 revisions of the McDonald Criteria. Lancet Neurol 2018; 17(2): 162–173.)  ^e^ American Psychiatric Association (2013) Diagnostic and statistical manual of mental disorders (5th ed.)  ^f^ Hakim et al. N Engl J Med, 1965; 2:307-27  Abbreviations: RRMS = relapsing-remitting multiple sclerosis; SPMS= secondary-progressive multiple sclerosis; YGTSS-TTS = Yale Global Tic Severity Scale Total Tic Score. | | | |
