# Supplementary material for: Phosphatidylcholine PC ae C44:6 in cerebrospinal fluid is a sensitive biomarker for bacterial meningitis
Source: J Transl Med. 2020 Jan 7;18:9. doi: 10.1186/s12967-019-02179-w (PMC6945415; doi:10.1186/s12967-019-02179-w)
Supplement: Supplementary file 2 — Additional file 2: Table S2. Demographic and clinical laboratory characteristics. [file 12967_2019_2179_MOESM2_ESM.docx]

| **Table S2.** Demographic and clinical laboratory characteristics^*^ | | | | | | | | | | | | | |  |
| --- | --- | --- | --- | --- | --- | --- | --- | --- | --- | --- | --- | --- | --- | --- |
|  | **DEMOGRAPHIC** | | | **BLOOD PARAMETER** | | **CSF PARAMETER** | | | | | | | | |
|  | **Sex [%]** | | **Age [years]** | **Leukocyte**  **[1000/µL]** | **CRP**  **[mg/L]** | **Leukocyte**  **[1/µL]** | **Protein**  **[mg/L]** | **IgG-Index** | **Lactate**  **[mmol/L]** | **Blood-CSF-barrier disruption [%]^§^** | | | | |
|  | **Female** | **Male** | **Median (range)** | **Median (range)** | **Median (range)** | **Median (range)** | **Median (range)** | **Median (range)** | **Median (range)** | **No disruption** | **Light** | **Moderate** | **Severe** | |
| **BacM**  **(n = 32)** | 47 | 53 | 51  (18-83) | 16.2 (6.5-48.4) | 100  (1-429) | 764.8  (4.3-18800) | 2106  (353-9900) | 0.65  (0.03-0.85) | 7.21  (1.82-21.94) | 3 | 19 | 19 | 59 | |
| **Borrelia**  **(n = 34)** | 47 | 53 | 48  (18-84) | 7.1  (3.4-13.7) | 1  (0.1-52) | 113  (7.7-1025) | 1125  (226-2471) | 0.8  (0.47-1.5) | 2.32  (1.41-5.29) | 23 | 18 | 41 | 18 | |
| **HSE**  **(n = 9)** | 22 | 78 | 56  (29-76) | 10.1  (1.8-12.8) | 3  (1-102) | 90.7  (16-723) | 1155  (524-2271) | 0.76  (0.50-1.32) | 2.31  (1.82-3.35) | 11 | 11 | 11 | 67 | |
| **VZV ME**  **(n = 15)** | 40 | 60 | 55  (13-80) | 6.9  (3.8-13.6) | 2  (1-128) | 38  (1.7-1536) | 649  (313-2048) | 0.56  (0.5-1.16) | 2.73  (1.49-5.5) | 21 | 36 | 29 | 14 | |
| **EntM**  **(n = 10)** | 40 | 60 | 32.5  (22-76) | 7.3  (4.0-14.0) | 4  (1-39) | 9.15  (0.7-619) | 518  (240-976) | 0.53  (0.47-0.63) | 1.88  (1.55-3.55) | 20 | 70 | 10 | 0 | |
| **VZV Fac**  **(n = 16)** | 56 | 44 | 64.5  (20-89) | 6.7  (4.1-13.2) | 2.5  (1-18) | 17  (0.7-800) | 503  (270-1485) | 0.54  (0.43-0.85) | 1.87  (1.44-4.24) | 50 | 31 | 19 | 0 | |
| **VZV Seg**  **(n = 14)** | 57 | 43 | 60  (49-79) | 5.9  (2.4-9.0) | 2  (1-15) | 2  (0.3-17.7) | 554.5  (273-880 | 0.54  (0.43-0.77) | 1.78  (1.42-2.08) | 54 | 38 | 8 | 0 | |
| **NMDA**  **(n = 8)** | 70 | 30 | 27  (19-69) | 6.5  (2.8-18.2) | 14.2  (1-37) | 20  (1.7-172) | 350  (254-480) | 0.62  (0.53-1.39) | 2.22  (1.38-2.64) | 75 | 15 | 0 | 0 | |
| **MS**  **(n = 17)** | 47 | 53 | 33  (23-58) | 6.5  (2.8-18.2) | 1  (1-23) | 7.7  (0.7-48) | 494  (309-663) | 0.79  (0.55-2.91) | 1.62  (1.31-2.37) | 53 | 47 | 0 | 0 | |
| **Bell's**  **(n = 11)** | 55 | 45 | 45  (22-83) | 8.3  (4.6-11.9) | 3  (1-31) | 2  (0.3-4.7) | 490.5  (309-829) | 0.47  (0.41-0.59 | 1.57  (1.24-2.22) | 55 | 45 | 0 | 0 | |
| **GTS**  **(n = 20)** | 10 | 90 | 38.5  (19-64) | 6.2  (4.7-8.7) | n/d | 1  (0.3-4.7) | 353.5  (228-624) | 0.52  (0.46-1) | 1.61  (1.43-1.90) | 80 | 20 | 0 | 0 | |
| **NPH**  **(n = 35)** | 43 | 57 | 68  (20-91) | 6.9  (3.7-11.7) | 1  (0.3-29) | 0.7  (0.3-11.3) | 404.5  (147-853) | 0.5  (0.42-0.69) | 1.67  (1.21-2.5) | 69 | 28 | 3 | 0 | |
|  |  |  |  |  |  |  |  |  |  |  |  |  |  | |
| ***P* value^#^ (all patient groups)** | **1.7e-01^§^** | | **9.2e-05** | **6.1e-09** | **5.4e-13** | **0.0** | **1.1e-16** | **7.3e-15** | **0.0** | **2.2e-16^§^** | | |  |  |
| ***P* value^#^ (inflamed vs non-inflamed)** | 0.484^§^ | | 0.135 | **0.006** | **0.008** | **0.0** | **1.7e-13** | **1.0e-11** | **6.3e-14** | **1.2e-13^§^** | | | | |
| ^*^ Adapted from ref. 4 (Sühs et al. J Infect Dis 2019; 220:127-138) with kind permission by the publisher.  ^#^ Calculated by Kruskal-Wallis test unless stated otherwise.  ^§^ Calculated by Chi-squared test. Bold numbers: *P* ≤0.05.  ^§§^ Patient-adjusted Q-albumin reference value = age/15 + 4. No disruption ≤ reference value, light ≤15, moderate ≤25, severe >25.  Abbreviations: BacM - bacterial meningitis; Bell’s - idiopathic Bell’s palsy; Borrelia - *Borrelia burgdorferi* neuroborreliosis; CRP – C-reactive protein; EntM - enterovirus meningitis; Gille de la Tourette syndrome; HSE – herpes simplex virus encephalitis; MS - multiple sclerosis; n/d – not determined; NMDA – anti-NMDA-receptor autoimmune encephalitis; NPH - normal pressure hydrocephalus; VZV Fac - facial nerve zoster; VZV ME – varicella zoster virus meningoencephalitis; VZV Seg - segmental zoster (shingles). | | | | | | | | | | | | | | |
